# Reflections on a career as graduate mentor—from baby steps at Wisconsin to today

**DOI:** 10.1093/jas/skad136

**Published:** 2023-04-29

**Authors:** Peter J Hansen

**Affiliations:** Department of Animal Sciences, D.H. Barron Reproductive and Perinatal Biology Research Program, and Genetics Institute, University of Florida, Gainesville, Florida 32611-0910, USA

**Keywords:** animal science, graduate education, physiology

## Abstract

Graduate education is an important aspect of the life of most academic scientists and a serious responsibility because it comes with the obligation to help students achieve their career and life goals. It can also be very fulfilling for the graduate mentor in terms of personal satisfaction and advancement of the research program. Learning to be a good major professor is an active process that depends on developing a formal framework of education and modifying that framework for each student based on past experiences and experimentation, advice from colleagues, and the individual personality of the student. Perhaps most important is for the graduate mentor to buy into the success and well-being of the student. Among the characteristics that a major professor could seek to instill in his or her students are critical and independent thinking, self-confidence, a thick skin, teamwork, laboratory skills and understanding, and the ability for hard work. Work to make science joyful by celebrating accomplishments, creating a fun environment in the lab, and stressing the societal value of science as compared to personal rewards or ambition.

## Teaser

Want advice on how to mentor graduate students? This paper, like all papers on graduate education written by Casida Award recipients, has some food for thought.

*A criticism of much science teaching is that it places too high a priority on ‘information’ transfer, and thereby engenders an uncritical attitude on the part of students. Students feel that there are so many facts which need to be learnt that there is no time for questioning, no room for scepticism. Yet the distinctive feature of scientific ‘knowledge’ is its impermanence: few practising scientists will not have had to modify their opinions quite substantially over, say, the last ten years. In short, science is not simply a body of ‘facts’, but an activity,... While we may derive a certain amusement from the apparent naïveté of our predecessors, we should forbear derision: our own views will doubtless suffer a similar fate.*

*T. B.* *Mepham (1987)*

## Baby Steps

**Figure 1.**
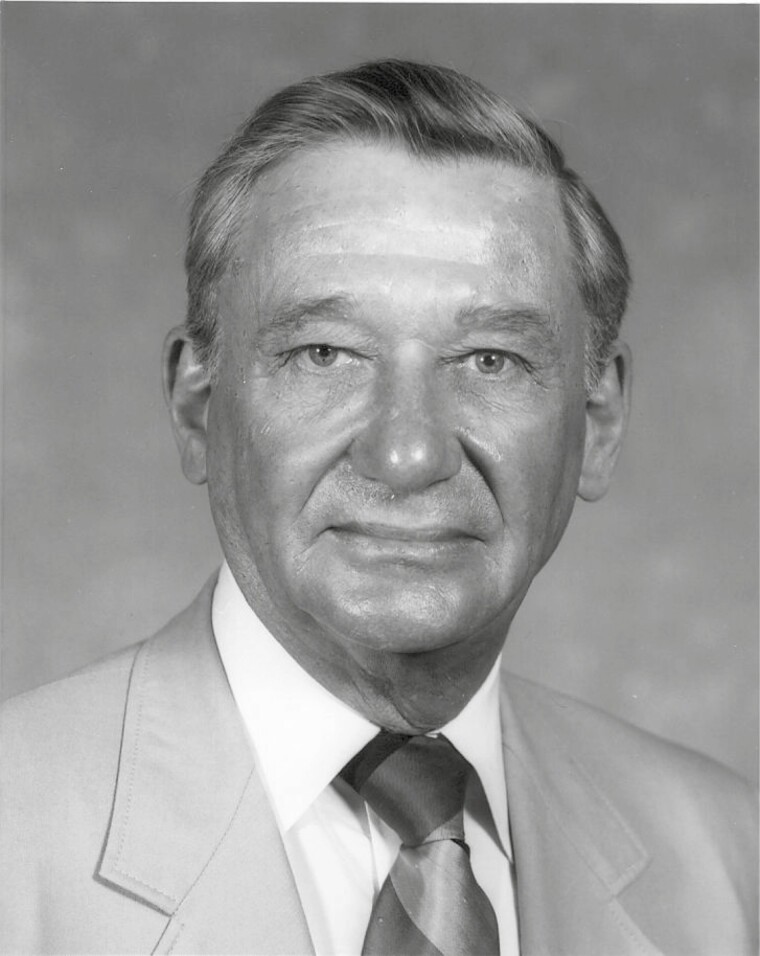
Edward R. Hauser (1916-2014) who was on the faculty of the University of Wisconsin from 1949 to 1988. Hauser was my major professor and imparted to me key principles of scientific discovery. He also got me to think, for the first time, about how I would approach mentoring my own graduate students after obtaining a faculty position. The photograph is from Wikipedia.

The person who did more than anyone to make me the scientist and graduate mentor I am today was Edward R. Hauser (Figure 1). This paper is dedicated to his memory and legacy. Growing up on a farm outside La Crosse Wisconsin, Hauser received the B.S. degree in Animal Husbandry from University of Wisconsin in 1938 and the M.S. degree from Oklahoma A&M University in 1939 after which he became assistant professor at Clemson College. His activities there were interrupted by a tour in the U.S. Navy during the Second World War where he served on the USS *Chincoteague* and saw action at Peleliu, Iwo Jima, and Okinawa. He returned to Clemson briefly after the war but then enrolled in a doctoral program at the University of Missouri under the supervision of Gordon Dickerson (himself a Wisconsin graduate) studying the genetics of boar sexual development.

Hauser returned to Wisconsin in 1949 as an assistant professor in the Department of Animal Husbandry. Although hired as a geneticist, Hauser came to Wisconsin with a strong interest in reproductive biology. He quickly became a collaborator and informal mentee of Lester E. Casida. Many of the graduate students he supervised in reproductive physiology were comentored with Casida. Hauser drank deep from the Casida philosophical well.

**Figure 2.**
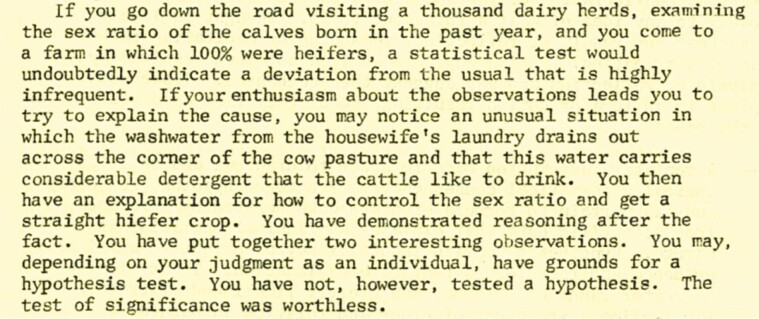
Portion of a mimeographed text written by Lester Casida to help with interpretation of journal articles. Here, Casida gives an example of reasoning after the fact. The entire document is reproduced in Supplemental File 1.

It is a tremendous honor to receive the L.E. Casida Award for Graduate Education. Casida had recently retired when I came to Wisconsin in 1978. He did come into the lab occasionally to confer with Dr. Hauser. He did not speak to us graduate students directly but his feedback regarding our projects would be conveyed to us through Hauser. Indeed, the philosophy of Lester Casida has been with me since my first days in graduate school when I was handed a mimeographed copy of a paper Casida wrote entitled “Some terms and ideas which may be of value in evaluating journal articles”. A small section of that paper is reproduced in Figure 2 in which Casida explains reasoning after the fact. The entire document is in Supplemental File 1. I well remember studying that paper and vowing never to make the kind of mistake highlighted by the case of the washwater affecting the sex ratio.

In those days, most of the animal reproduction faculty at Wisconsin and throughout the country had been imbued with the ideas regarding scientific practice that Casida taught. Among these were formation of a philosophical structure for using science to develop new knowledge, the centrality of the scientific method, reliance on statistics for unbiased hypothesis testing and for describing biological relationships between variables, and development of an understanding of how to use techniques and learn new ones (Casida, 1966).

It was Ed Hauser who imparted the Casida philosophy to me and who became a role model of what I still perceive as the virtuous scientist. Hauser was an acolyte of the philosopher and author Ayn Rand and believed that one’s satisfaction with oneself should not be put into the hands of others. He did science to understand nature and not to receive awards or other accolades. The value of doing science to Hauser was the discovery of knowledge rather than personal success. Among the other lessons he taught me was to ask a biological question and let the experiment provide the answer rather than fitting the results to the desired answer. Hauser taught me to not be swayed by authority (the voices of distinguished scholars) but only by experimental results. He also instructed me to be patient (something I am still working on).

Dr. Hauser gave me much practice in thinking for myself. I vividly remember going into his office each time I wished to start a new experiment. He would send me to the blackboard to sketch out the experimental design. He would gently point out weaknesses or problems in the design and send me back to the lab to think of a solution. He never told me how to design the experiment but rather let me develop the ideas myself. I am pretty sure that most times we ended up doing an experiment close to what Hauser wanted done in the first place but the process with which we derived the experiment taught me how to formulate experiments.

Even though I was only a graduate student, Hauser encouraged me to think about how I would educate my graduate students once I became a faculty member. He believed that his greatest legacy was the students he helped form into scientists. At the time, I dismissed the idea that this would be the case for me when I had my own lab. I recognized the importance of graduate education but I was in love with the idea of scientific discovery. As I have gotten older, I have come to realize that there is much merit to his views.

There are some lessons for graduate mentors in this section of personal reminiscence. The first lesson, and the most important, is that, for better or worse, graduate mentors have a powerful impact on their graduate students and one should take on the role of graduate mentor with that realization. The second lesson is to actively teach students a philosophical structure that can be used to design, conduct, and interpret experiments. I depend greatly on the basic processes of the scientific method I was taught as a graduate student (observation, formulation of a hypothesis, designing and carrying out an experiment to test the hypothesis, and appraisal of the hypothesis after interpreting experimental results) as well as on Karl Popper’s views on empirical falsification of scientific ideas (Popper, 1959). The third lesson is to get your students to start thinking for themselves—do not be afraid to have them contradict you or express uncommon or even heretical views.

The final lesson is to encourage your students to contemplate how they might function as graduate mentors. In my own case, despite prior thinking about the topic, I was still unprepared to be a good graduate mentor when my first students arrived in the lab. I was 28 yr old and some of my first students were older than me. Nonetheless, I am sure that the effort I made in developing an intellectual framework for how I wanted to approach graduate mentoring increased the slope of my learning curve. I hope this paper can do the same thing for the reader by providing some ideas that may prove fruitful when the opportunity for graduate mentoring presents itself.

## How to Learn How to Be a Good Major Professor

I just stated that you should have an intellectual framework to guide your efforts as a graduate mentor. In other words, think about how you would go about it. Having done so, test your ideas on your graduate students. You might find that some of the practices you have developed in your mind are very effective in practice while others fall flat. Retain those that work and discard those that do not. Not all students are alike so what works great with one student will not work with another. Try things out, experiment, and adapt.

Do not go into the world of graduate education alone but rather lean on trusted colleagues for advice and insight. In my own career, I have often sought advice from Dr. William W. Thatcher, who is himself the 1997 recipient of the Casida Award. For many years, my office was right next to Bill’s. I often went there for advice on how to deal with difficult situations with a student. I also have learned much from watching other faculty members interact with graduate students. Some were good at it and some were bad but I could often identify specific practices that seemed to work and others that should be avoided. I like observing people and see how they live their lives, not only scientists but people throughout my daily experiences. I study them and frequently borrow or discard ideas that might further my career in science.

I am a fanatical reader of history and biography. I read for many reasons but one of which is for the life lessons that I can accrue. For example, the animal geneticist C.R. Henderson spoke to me indirectly through his biography (Van Vleck, 1991) where seven pieces of advice Henderson gave to young scientists at a meeting in Kyoto were presented (Table 1). When reading a biography of Louis Pasteur, I recognized that, although he was a great scientist, his penchant for control and secrecy, even towards his own lab personnel, probably made him an inadequate teacher of young scientists (Geison, 1995).

**Table 1.** Some advice to young scientists from C.R. Henderson given in an address at Kyoto University, December 16, 1985 (Van Vleck, 1991)

1. Study methods of your predecessors.
2. Work hard.
3. Do not fear to try new ideas.
4. Discuss your ideas with others freely.
5. Be quick to admit errors. Progress comes by correcting mistakes.
6. Always be optimistic. Nature is benign.
7. Enjoy your scientific work. It can be a great joy.

## Buy Into Your Students

To a large extent, taking on a graduate student is a selfish decision. After all, the graduate mentor wants that student to work hard and with passion and to help develop the research ideas that the mentor is exploring. Every academic scientist wants to identify and recruit the student who will be a star—where everything they do in the lab works, where they help their fellows willingly, and where they bring new ideas to the lab. Fair enough since the major professor must acquire funding to support the student and many hours of mentoring to bring the student along. What one should never do though is lose sight of the student’s own goals which include at a minimum graduation and progression towards an independent career. Casida himself once warned that when considering graduate education “you must always remember that you are playing with people’s lives” (Inskeep, 2000).

Although we want students to be productive, pushing students to optimize their output at the expense of everything else can be destructive of lab morale and mental health. Go onto Twitter and look up tweets for accounts like @hapyresearchers or hashtags such as #phdlife. Graduate students often feel like they are just cogs in the wheel of science and are unvalued as scientific companions. These students will not be good ambassadors of their program and will sometimes be unprepared for the slog ahead as they seek permanent employment and research opportunities.

It is not uncommon to see faculty members retain outstanding graduate students in the lab past the time when they have completed a body of work sufficient for the degree. To do so is unethical and a betrayal of the trust that the student and their family have placed in you as a mentor. More common is the lazy or uninterested graduate mentor who takes on a student but does not give him or her the attention and resources necessary for the student to thrive. “I’m too busy” is the all-too common refrain of faculty who will not read drafts of theses or papers or who do not acquire the resources to support the lab. If you are too busy, do not take on a student you cannot adequately mentor.

What I suggest is to buy into each student—your success and life satisfaction depends upon theirs. Do what you can to provide each student with the opportunity to grow as a scientist. Study each of your students and see what motivates them. The first thing I had to learn about graduate education when I started as an assistant professor was that my students were not all younger versions of me. Often, they had different goals, work ethic, and attitudes towards science. Once I learned that, I realized I could tailor each student’s experience to meet their goals while also exploiting their strengths to advance the mission of the lab. Students became happier and more productive. I also learned from Bill Thatcher that I should not be disappointed if each student was not the next scientific rock star. Bill often reminded me that I had succeeded if I had helped each graduate student reach their full potential no matter what that potential was.

Finally, don’t judge the competence of your students too early in their career. Many times, I have seen students struggle to understand the fundamentals of scientific inquiry until, one day, midway or late in their graduate program, the lights come on. This has been the case for many a prominent scientist. Indeed, Robert Waring Darwin once said to his son Charles Darwin “You care for nothing but shooting, dogs, and rat catching, and you will be a disgrace to yourself and all your family” (Darwin, 1887). Sometimes a person who appears to be a bad fit for a life of science is just wrestling with themselves to find the best way forward to succeed in science.

## Some Characteristics to Foster in Graduate Students

If, when your student graduates, he or she has many of these characteristics, you can feel satisfied that you have been a successful graduate mentor.

### Critical thinker

Meet with your students often and use those interactions as an opportunity to engage in critical thinking. I have a lab meeting each week where, after discussing issues related to laboratory management, one student leads a discussion of a recent research paper or one of their ongoing or planned research projects. The focus on paper discussions is not on the facts to be derived from the paper but on the process by which scientific conclusions were derived. “Practice makes perfect” is an old proverb and discussion of research papers is a way to practice experimental design and interpretation without all the expense and time of a real experiment.

I also set aside one hour a week to meet with each student individually. I use this time to discuss research progress, plan future experimentation, discuss other issues faced by the student, and to try to understand each student more deeply.

### Independent thinker

**Figure 3.**
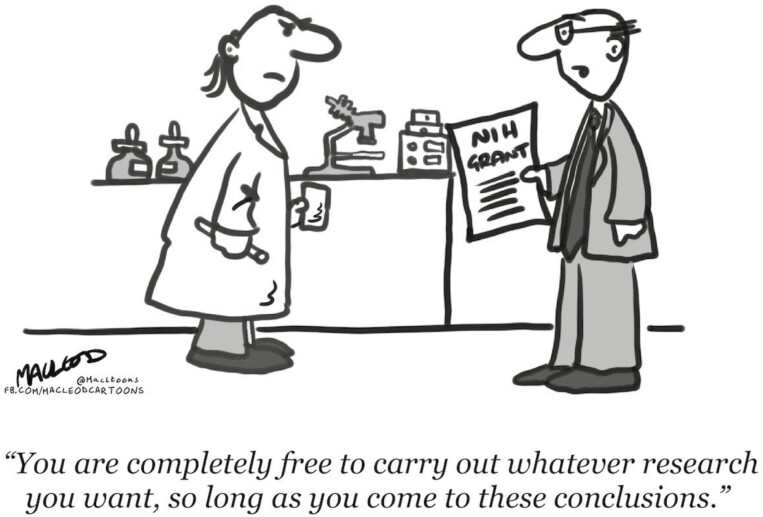
How not to foster independent thinking. The cartoon is reproduced by permission from James MacLeod, University of Evansville (https://www.facebook.com/MacLeodCartoons/).

The reality of science today is that most research is funded by extramural grants and it is important that the research objectives of the grant be pursued. This situation means that there are constraints to each member of the laboratory in terms of research topic and direction. Nonetheless, it is possible to develop a student’s research program so that they can contribute intellectually to the direction the research takes, particularly once research findings accumulate and thinking about the research necessarily changes. It is important to encourage each student, and indeed each member of the laboratory and yourself, to interpret the results of experiments in an independent manner and not to try to force interpretations of research results to fit a pre-ordained hypothesis that was the basis for the grant (Figure 3). Students should not be made to be apprehensive or fearful about challenging any idea, even if that idea is the basis for the laboratory’s funding. One of the great features of scientific pursuits is the ability to rethink well-established ideas and develop new ones closer to the truth.

### Self-confident

In graduate school, I had a friend who used to work in a laboratory at a midwestern Land Grant University. According to my friend, everyone in that lab was terrified of making a mistake because of the fury that would be exhibited by the principal investigator. One day, a student dropped a flask which broke on the floor and, such was their fear of the professor, someone screamed! This is the not the kind of environment in which to build self-confident scientists. Rather, work to promote a milieu in which fear of failure has been banished.

People vary in their degree of self-assuredness, of course, but many come to the laboratory intimidated by one or more aspects of the scientific process, whether it be performing specific procedures in the laboratory, working with animals, presenting papers at lab meeting, writing, or expressing their opinion about a scientific issue. It can be difficult to build confidence in one’s abilities because things constantly go wrong in science—techniques do not work, experiments are not repeatable, ideas are criticized. Unless a student gains trust in his or her own abilities, he or she is likely to shirk from activities that might bring failure. For example, many students never seem to spend much time at the lab bench. Often, it is because they are afraid to fail.

Conceptually, the graduate mentor’s role in building self-confidence is easy. Do not get mad or disappointed in your students when a technique does not work, an idea is misguided, or a manuscript is confusing. Work with the student to find solutions to the problem and encourage them to try again. This all sounds easy but faculty are themselves under pressure to perform. It can be hard to say “Don’t worry - $10,000 of RNA samples just went down the drain because you did not snap-freeze the samples”. Nonetheless, constantly impress on each student that failure is normal and that criticism of ideas and practices is not personal but a way towards the truth.

### Thick-skinned

In some ways, I view science as a solitary occupation—me vs. the research problem. I do a lot of my thinking while driving, walking, or in the shower. Nonetheless, some of my best research ideas came about because some other scientist brought me to a realization I might not otherwise have. In 1993, Nobelist James Watson gave a talk at Cold Spring Harbor in honor of the 40th anniversary of the discovery of the double helix. At that talk, Watson said “To succeed in science, you have to … always turn to people who are brighter than yourself. …It’s to go somewhere beyond your ability and come out on top” (Watson, 1993). He is right and we will be more likely to succeed at a high level if we surround ourselves with outstanding people than with people who are mediocre scientists or who are loath to criticize others. 

If you can accept the need for serious scrutiny by others (and you should), teach that value to your students. Get your students (and yourself) exposed to scientists who will challenge and provoke. Pick committee members who will provide students with the opportunity for constructive engagement. Do not limit your student’s exposure to other scientists (this is a surprisingly-common phenomenon). Encourage your students to reach out to other scientists at their home institution or elsewhere. Scientific conferences are a great place to do so.

### Team player

*“*One of all and all for one” (*Un pour tous, tous pour un*) was the motto of Alexandre Dumas’s Three Musketeers. Adoption of that slogan will make each of your students a more ­valuable partner, whether in the laboratory, the university faculty, or in the scientific societies that still underpin much of scientific practice today. Working together towards a common goal, where everyone contributes and where everyone wins, is one of the most effective approaches to success.

Students learn to value teamwork by seeing it effectively executed in practice. Fostering of teamwork by the major professor requires the mentor to be loyal to each of his students. Each student came to your laboratory to pursue their own life goals and he or she has a right to expect to be given every opportunity to pursue them while still working for the good of the laboratory. Every student should be treated fairly and with respect. They are not children but college-educated scientists. Treat them like the professionals they are. Motivate them by focusing on the scientific problems addressed in the lab and the satisfaction to be gained by solving those problems. Do not tell them they need to publish a paper so you can get a raise or that they need to work harder so you can get tenure. Lastly, practice what you preach. If you tell your students that you expect them in the lab 7 d a week (don’t tell them that), then you show up 7 d a week.

### Laboratory wizard

Keith Inskeep, the 1998 recipient of the Casida Award, wrote that “Scientists must guard against inventing problems to utilize existing techniques, and develop the in-depth thinking that will allow our students to invent techniques to solve the problems” (Inskeep, 2000). Right on Keith! One reason why scientists do experiments based on what techniques they know rather than on where the science leads them is because they are limited in technical ability. How often have we seen scientists relying on the same techniques they learned in graduate school 20 yr later?

Instilling a love of working in the laboratory should be a priority for the graduate mentor. One way to achieve this was addressed earlier—avoid any opprobrium associated with failure to get a technique to work. If need be, reach out to collaborators or other colleagues to find the key to getting a technique reduced to practice by the student. Importantly, teach students to ask themselves what is the chemical or physical basis for the technique they are working on? Why are proteins often dissolved in phosphate-buffered saline containing bovine serum albumin? Why does phosphate-buffered saline have the formulation it does? Why do you add a detergent to buffers for immunochemical techniques? After several years of asking and answering questions like these, the student will be well on his or her way to being a master of scientific technique, who can design techniques on the fly rather than relying on menus written down by someone else.

### Hard worker

One issue everyone in academia is wrestling with is work-life balance—how to have a successful and satisfying academic career without giving up family, friends, and outside activities. I don’t have any easy solutions to the problem. One reality is that science is incredibly competitive and there will always be someone you are competing with who is working nonstop. You can work “smarter not harder” but somewhere you have a competitor working smarter and harder. You cannot be a world-class scientist without putting in long hours. Certainly, you do not have to be a world-class scientist to be successful. Regardless, working out work-life balance starts in graduate school and the mentor should be cognizant of how to ensure that their students are sufficiently committed to their research but still have time for family and outside activities. As outlined in the next section, one key is to foster an environment in which the process of science is joyful.

## Some Tips for Making Science Joyful

As has been alluded to several times in this paper, science can be stressful, grueling, frustrating, and defeating. It can also be wonderful and, occasionally, even exhilarating. Both these aspects of science are encapsulated in a letter written by Theobald Smith, who cured Texas Cattle Fever, in which he wrote “to those who have the urge to do research and who are prepared to give up most things in life eagerly pursued by the man in the street, discovery should come as an adventure rather than as the result of a logical process of thought” (Zinsser, 1987). Foster feelings of adventure and joy as a laboratory director and you will have a happy and productive laboratory. Here are a few tips for doing so.

### Celebrate the triumphs—great and small

**Figure 4.**
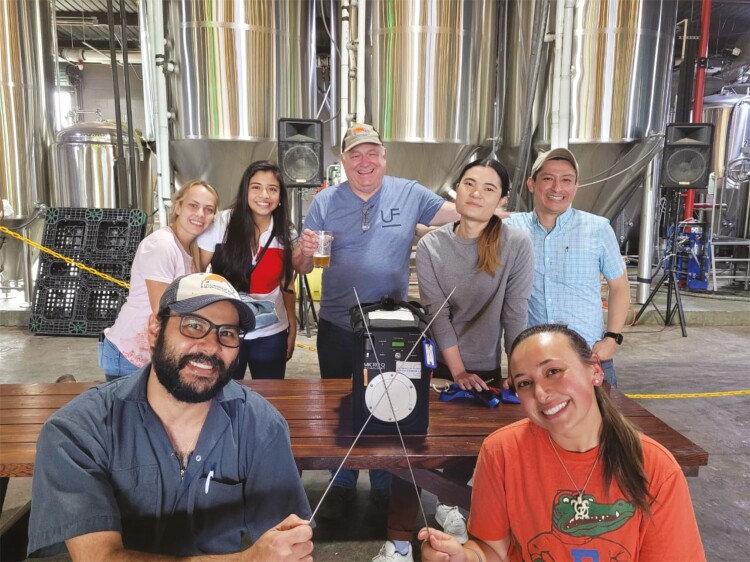
An example of how to celebrate the little successes in science. This photograph was taken at First Magnitude Brewery (Gainesville, Florida) in April 2021 following the last of a large series of embryo transfer experiments conducted throughout the state of Florida. Posing with a portable incubator and two embryo transfer pipettes (l-r) in front are Thiago Amaral and Lané Haimon and in the rear Tatiane Maia, Camila Cuellar, myself, Surawich Jeensuk and Eliab Estrada-Cortés.

My doctoral student Morgan Peltier would often walk into my office to announce “I’ve had a Eureka moment”. He did so while being cognizant of the Greek polymath Archimedes who shouted “I have it” when he discovered the principle of buoyancy. Morgan recognized the thrill of scientific discovery and reveled in each one he made even if it was small. That was a lesson for me. Accordingly, I try to seize those opportunities to cry “Eureka” myself whenever possible and to celebrate each of my student’s successes big or small (Figure 4). Doing so takes some of the sting out of the all-too-common situation of failure.

### Build a culture around the nobility of science

No one goes into science so their major professor can get a grant. Some become scientists because of its purity and potential for good. Francis Bacon said it as early as the 16th Century when he wrote “Lastly, I would address one general admonition to all; that they consider what are the true ends of knowledge, and that they seek it not either for pleasure of the mind, or for contention, or for superiority to others, or for profit, or fame, or power, or any of these inferior things; but for the benefit and use of life; and that they perfect and govern it in charity” (cited by Klein and Giglioni, 2020). Science gave us the atom bomb, to be sure, but science also is used every day to better the human condition. As an agricultural scientist, I am faced with the compelling need to make science practical. It is great motivation to let a young scientist know that what they are doing will eventually contribute to some new practice, procedure, or device that will make for a better world.

### Have fun

**Figure 5.**
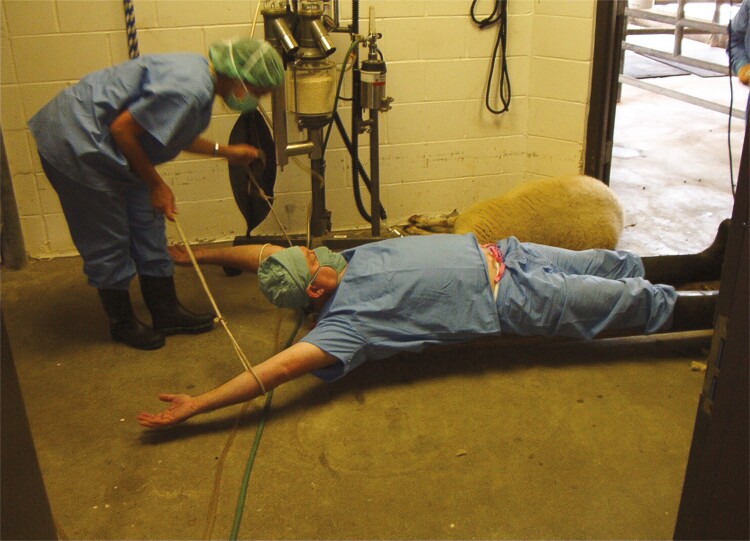
An example of research being fun! This photograph, taken in the Animal Sciences surgery at the University of Florida circa 2004, shows the author demonstrating for Maria Padua the proper way to tie a sheep on a hurdle in preparation for surgery.

As the last paragraph illustrates, science is grand. It can also be fun! The great reproductive biologist Carl G. Hartman, who among other things was the first to describe the bovine egg (Betteridge, 2007), said that “Research should spell fun” (Hartman, 1967). Indeed it should! My advice would be to try to create an environment where people like each other’s company and form friendships, where they feel comfortable enjoying themselves, where gentle humor is common, and where people like to play. As a graduate mentor, use your sense of humor in a positive way and don’t take yourself too seriously (Figure 5). Socialize with your students in moderation so they know you like them and value their company but let them live their own lives and have time away from the laboratory.

## Final Thought

Mentoring can be an intimidating experience—those are real-life people you are dealing with who have not read the manual of how to respond to their major professor in a manner pleasing to him or her. The things emphasized here often work for me but there are certainly other ways to approach graduate education. Perhaps the most important piece of advice I have proffered is the one that asks you to buy into your students. If you invest the time and think about what you are doing, things will usually work out in the end for both student and professor.

## Supplementary Material

skad136_suppl_Supplementary_File
