# Supplementary material for: Reflections on a career as graduate mentor—from baby steps at Wisconsin to today
Source: J Anim Sci. 2023 Apr 29;101:skad136. doi: 10.1093/jas/skad136 (PMC10257352; doi:10.1093/jas/skad136)
Supplement: skad136_suppl_Supplementary_File [file skad136_suppl_supplementary_file.pdf]

Some Terms and Ideas Which May Be of Value
in Evaluating Journal Articles

Science -- Knowledge covering general truths or the operation of general laws, especially as obtained and tested through scientific method.

Scientific Method -- Principals and procedures for the systematic pursuit of knowledge involving recognition and formulation of a problem, the collection of data through observations and experiment and the formulation and testing of hypotheses.

Idea -- A plan for action; an indefinite or unformed conception; a formulated thought or opinion. Idea may apply to a mental image or formulation of something seen or known or imagined. Concept may apply to the idea formed by consideration of instances of a species or genus or more broadly to any idea of what a thing out to be. Thought is likely to suggest the result of reflecting, reasoning or meditating rather than of imagination. Notion suggests an idea not much resolved by analysis or reflection and may suggest the accidental. Impression applies to an idea or notion resulting immediately from some stimulation of the senses.

Hypothesis -- A tentative assumption made in order to draw out and test its logical or empirical consequences. The terms hypothesis, theory and law pertain to formula or statements derived by inference from scientific data that explains a principal operating in nature. Hypothesis implies insufficiency of presently attainable evidence and therefore a tentative explanation. Theory implies a greater range of evidence and greater likelihood of truth. Law implies a statement of order and relation in nature that has been found invariable under the same conditions.

Thesis -- A position or proposition that a person advances and offers to maintain by argument.

Experiment -- An operation carried out under controlled conditions in order to discover an unknown effect or law, to test or establish a hypothesis, or to illustrate a known law.

The following statements were abstracted from a talk given by Dr. L.E. Casida:

"Reasoning after the fact is one of the most important steps in experimental research. I shall define the phrase 'reasoning after the fact' as arriving at a decision, an interpretation, a course of action or an attitude from examination of assembled observations. It is the formulation of a generalization that best fits the known facts. A large part of historical research involves reasoning after the fact. History then becomes somebody's interpretation of what happened and how and why.

An antonymic phrase, reasoning before the fact, is defined as an expectation following upon reasoning after the fact. It includes formulation of a hypothesis to test the generalization. A prediction is made on the basis of the hypothesis. An experimental design is developed to give opportunity for the hypothesis to express itself as predicted.

Reasoning before the fact is pointed at testing the hypothesis. It should lay down as precisely as possible treatment, appropriate control, endpoints to be measured and comparisons to be made. Emphasis is placed on the comparisons to be made.

The protection of an investigator by a test of significance of his results is available only if he plays the game fairly at this point. He is given methods for estimating the probability of differences as great or greater than his having been due to chance alone. He can determine the probability that comparable samples drawn from a homogeneous population might have shown differences as great or greater than his. A given probability level can mean that level, however, only if a decision to compare two samples has been made completely independent of existing differences between the samples.

If you go down the road visiting a thousand dairy herds, examining the sex ratio of the calves born in the past year, and you come to a farm in which 100% were heifers, a statistical test would undoubtedly indicate a deviation from the usual that is highly infrequent. If your enthusiasm about the observations leads you to try to explain the cause, you may notice an unusual situation in which the washwater from the housewife's laundry drains out across the corner of the cow pasture and that this water carries considerable detergent that the cattle like to drink. You then have an explanation for how to control the sex ratio and get a straight hiefer crop. You have demonstrated reasoning after the fact. You have put together two interesting observations. You may, depending on your judgment as an individual, have grounds for a hypothesis test. You have not, however, tested a hypothesis. The test of significance was worthless.

What should be considered acceptable reasoning after the fact? In opening a new area of inquiry, little or nothing is known as to what to expect. There is little basis for formulating hypotheses. There are few generalizations. Inquiry must start from keen observations and from attempts to find associations among characters of unknown importance as starting points.

Something may be tried in an attempt to disturb the equilibrium of characteristics and samples of the population and then as many things as possible observed for affects and apparent associations of affects. Mortality, in attempts to verify such associations in specifically planned experiments,is likely to be high (the association between washwater and sex ratio in dairy herds). If general knowledge of the field is worth anything, hypotheses growing out of a body of knowledge are more likely to stand up than those which just happen to be noticed and for which no known theoretical cause is suspected."
